# Assessing the Expression of Emotions in Breast Cancer Survivors during the Time of Recovery: Perspective from Focus Groups

**DOI:** 10.3390/ijerph19159672

**Published:** 2022-08-05

**Authors:** Leticia Sanchez, Nelida Fernandez, Angela P. Calle, Valentina Ladera, Ines Casado, Enrique Bayon, Isaias Garcia, Ana M. Sahagun

**Affiliations:** 1 Department of Nursing and Physical Therapy, Faculty of Nursing, University of Leon, 24071 Leon, Spain; 2 Department of Biomedical Sciences, Institute of Biomedicine (IBIOMED), Faculty of Nursing, University of Leon, 24071 Leon, Spain; 3 Department of Basic Psychology, Psychobiology and Methodology of Behavioral Sciences, University of Salamanca, 37005 Salamanca, Spain; 4 Department of Electrical and Systems Engineering. University of Leon, 24071 Leon, Spain

**Keywords:** breast cancer, focus groups, information needs, long-term care, survivors, women’s health

## Abstract

Breast cancer has major public health implications, as it is the most frequent malignant tumor and the leading cause of cancer death in women. Survivors have many needs, including strategies to cope with the associated distress. We explore whether focus groups are useful for nurses to obtain information about the emotional state of breast cancer women, and develop strategies for coping with the stress that this disease entails. A qualitative study was carried out, involving 25 focus groups with 83 women treated for breast cancer, recruited from the local Breast Cancer Association (ALMOM). Four open-ended questions were employed, and 60-min discussions were carried out. They were transcribed, analyzed, coded, and the themes identified. Four major themes emerged, including “complex emotional evolution”, “emotional isolation”, “lack of information” and “inability to give advice”. Women admitted that this disease had been a stressful factor for them, causing emotional (anxiety, irritability, anger or guilt) and cognitive disorders (confusion, lack of concentration, forgetfulness). The use of focus groups in breast cancer survivors allows nurses to evaluate the expression of emotions in these women, and collect and share information about their feelings, thoughts and experiences, so that survivors can cope more easily with the stress related to their illness.

## 1. Introduction

Breast cancer is the most common malignancy, and the leading cause of cancer-related death among women. Approximately 2.1 million women have been diagnosed with breast cancer worldwide, and more than 600,000 died in 2018 [1]. In Spain, it accounted for nearly 28% of new female carcinomas in 2015 [2].

Cancer has its own connotations in human beings, affecting patients and their families, as well as those health professionals involved in their care, and the population in general, who wish to be free of it [3].

In women with breast cancer, the quality of life varies according to the treatments followed [4], their psychological characteristics [5], and the efficacy of the psychological interventions implemented [6,7], among others. Despite the importance of these factors, the emotional dimension of this disease and its influence on treatment and recovery are not a priority in the existing studies. Among those psychological and social responses that cancer patients experience, anxiety, depression, lack of energy and sexual dysfunctions are frequently mentioned, as well as loneliness and problems at work [8,9,10,11,12]. Moreover, the breast is an organ with an enormous cultural, psychological, sexual and affective influence, as it is one of the physical elements of women’s sexuality [13]. Fatigue is also a common and distressing symptom that may influence the performance of daily life activities and persist once breast cancer has been overcome. This is a multidimensional symptom that affects physical and mental states, as well as decreases motivation in breast cancer women [14].

To achieve a holistic approach to the treatment of breast cancer women, it is essential to work out the perceived emotions in these patients, to provide them with coping strategies. Nurses play an essential role in caring for and supporting these patients, as there is no clear support from other institutions or adequate financial support. Although several organizations or associations may be involved in the care of these patients, most of them do so on a voluntary rather than professional basis [15]. On the other hand, it should also be noted that one of the most pressing concerns for those patients who have overcome breast cancer is their successful return to work after treatment, taking into account their difficulties related to the physical appearance and, of much more importance for them, to their psychological and emotional problems [16].

Consequently, given the lack of information on the emotional dimension in breast cancer survivors, we have conducted a qualitative study to characterize their feelings and fears, and to evaluate if focus groups help women to cope with breast cancer. Thus, the aim of this study was to assess if taking part in focus groups and expressing emotions is useful to define the emotional state of women and to facilitate coping with the stress related to breast cancer, as well as to identify their main concerns and implications for future psychological intervention guidelines when treating them. The research question guiding this study was as follows: “*Are focus groups useful for nurses both as a method to obtain information about the emotional state of women with breast cancer, and to develop coping strategies with them?*”.

## 2. Materials and Methods

### 2.1. Study Design

A qualitative study with focus groups was carried out in women diagnosed and treated for breast cancer. Focus groups are considered an appropriate qualitative data collection method for a grounded theory approach [17], as they encourage dynamic and lively group interaction, exchange of thoughts, and reflection. The CASP checklist for qualitative studies has been followed throughout the study [18].

### 2.2. Participants and Data Collection

Participants were recruited among members of the local breast cancer association (ALMOM). Interested people were invited to contact the research leader (first author). Focus groups were then planned once several members of this association agreed to participate. Of the 112 eligible breast cancer patients that belong to ALMOM, 83 (74.1%) agreed to take part in this study.

A total of 25 focus groups with 3–4 women were randomly organized. Dates for the groups were established depending on the availability of participants and the room in the ALMOM facilities. Once confirmed, women were phoned 1–2 days before the session to remind them the event. Data collection took 12 weeks.

We used open-ended questions, as they allow patients to give more easily their opinion about their disease, facilitating the exchange of opinions and feelings. A guide was specifically carried out by the research team after a literature search [19,20,21]. Three questionnaires were also reviewed to design the guide [22,23,24]. After several discussions within the research group, a consensual agreement was achieved on the information to be collected, and according to the objective of the study. Four open-ended questions were then set. Those questions were as follows:

*1.*  *Could you differentiate the emotional phases you have gone through during your illness?*
*2.*  *Please, identify those people who were part of your more immediate social environment during your illness and the role played by them. Who has been your major support?*
*3.*  *What decisions have you made about your illness? Could you describe those strategies you have used to cope with it?*
*4.*  *You have to give advice to women who start experiencing breast cancer, what would you tell them?*

Questions were tested in a pilot group, to verify if discussion was adequately promoted. Data from this pilot focus group were also included in the study, as only minor revisions to the guide were made.

Focus groups were conducted by experienced moderators (research leader and one of the other nurses taking part in this study). An introduction was first given about the purpose and procedures to follow. Moderators informed women on their voluntary and confidential participation, encouraging them to share freely feelings and personal experiences. Participation and discussion were facilitated, ensuring the natural fluidity of conversation in a friendly atmosphere, and the answer to all the issues raised. The psychologist of the team also took part in the focus groups, to ensure that a restrictive approach was not created, allowing women to express themselves freely, providing the time and the necessary elements to do so. Discussions were ended after achieving data saturation and no further ideas were provided by the participants [25]. No data obtained in the focus groups were disregarded in data analysis. Sessions were approximately 60 min in length, and were audio-taped. Field notes were also taken by the second co-moderator. Participants also fulfilled a brief demographic and clinical questionnaire, and signed an informed consent. No other persons except the research members and participants were present.

### 2.3. Data Analysis

Focus groups were assessed using qualitative content analysis with an inductive approach [26]. Initially, discussions were transcribed and assessed. Transcriptions were read several times independently by the authors for accuracy, and field notes were used to complete information. After that, the research group members worked in pairs to provide codes without predetermined categories. Broader categories and preliminary themes were then established. Any discrepancy in coding were discussed in a weekly meeting, until a consensus was reached. Finally, the entire research group discussed and reformulated information, and a consensus was reached about the prominent themes. All members of the research group contributed and participated in the final analysis, increasing the trustworthiness of the analysis with respect to consistency between the data presented and the findings.

### 2.4. Ethical Considerations

The study followed the ethical principles of the Declaration of Helsinki. The study protocol was approved by the Research Ethics Committee of the University of Leon (ULE0092015), as well as the Review and Psychological Boards of ALMOM. Participants received verbal and written information about the study and were assured that their participation was voluntary, and that they could withdraw from the study at any time, without giving a reason. They provided full informed consent. Moreover, oral informed consent was obtained before data collection started. Data were coded in order to ensure anonymity and confidentiality throughout the analysis.

## 3. Results

The characteristics of the 83 participants are shown in Table 1. Most of them (77.1%) were between 40 and 59 years old (52.5 ± 8.1 years). In addition, 56.6% were married or had a partner, and mostly had children (80.7%). One third had secondary (37.3%) or higher education (33.7%), and almost half were employed (47.0%). The length of time in survivorship was 4.1 ± 2.9 years, ranging from less than 1 year to 15 years. Almost all participants (95.2%) reported that they underwent surgery and chemotherapy, and 74.7% radiotherapy. The most common diagnosis was stage II (83.1%). Regarding the current situation of disease, more than a half had no recurrence, and intervals between reviews were mostly between 6 months or 1 year (Table 2).

### 3.1. Major Findings

**Figure 1:**
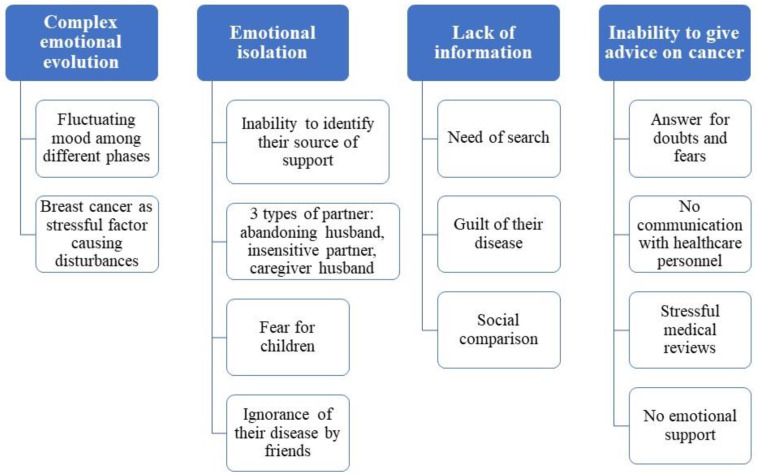
A framework of the themes and subthemes discussed.

Team discussions identified four prominent themes from the participant quotes, including “complex emotional evolution”, “emotional isolation”, “lack of information”, and “inability to give advice to other women”. All of them have been identified and summarized with the related subthemes in Figure 1, and described below. Quotations are also included to illustrate the findings.

#### 3.1.1. Theme 1: Complex Emotional Evolution in Breast Cancer Survivors

At first, when they were asked about those emotional phases that they had gone throughout since they became ill, there was a brief silence that quickly became a whirlwind of ideas, in which practically all participants agreed. They explained that their mood has evolved over time, fluctuating among several phases, not always in the same order.

**Denial**: They were not able to recognize such a traumatic situation. Many of them stated that “*they have behaved as if nothing is happening”*.

**Self-defense**: Once they were aware that there was a problem, they attributed it to their immediate social environment and not to themselves. They said the following statements:

*“What will my children think?”*

*“What a situation for my husband!”*

*“My mother is so old”.*

**Setback**: At that moment, they felt a change in their lives, becoming dependent people with childish attitudes against difficult situations.

*“It’s not fair that this should happen to me”.*

**Aggressiveness**: As the previous attitude did not imply constant emotional care, it was changed into aggressiveness, especially with those who might harm them. They specifically referred to healthcare professionals in the following ways:

*“Healthcare personnel do not treat me as I deserve”*

*“Health professionals do not understand me”.*

**Reactive training**: As they realized that aggressiveness was not the way, they took a different attitude, becoming independent from their social environment. They explained this with the following statements:

*“I don’t need anything from anyone”.*

*“I’m an adult and self-sufficient person”.*

**Withdrawal**: They defined this phase as one of the hardest moments for them. They decided to withdraw into themselves with the sole purpose of “*not disturbing* and *going unnoticed*, *perhaps even for cancer”*.

**Projection**: They often thought that everything would be different. Then, they tended to project themselves on someone (usually in a nearby woman) who “*is successful and healthy*” (not sick).

**Rationalization**: They tried to find an explanation for what was happening. They decided to adapt their desires, needs and feelings to what their social environment considered appropriate, as a way to avoid any criticism from “*the others*, *to be in the right dynamic*”.

**Compensation**: This was the moment when they thought their lives were a failure because of the disease they were experiencing. They said the following statements:

*“Everything I’ve done so far is useless”*

*“My life is meaningless”.*

**Emotional distance**: They described their experience with breast cancer in a distant way, as something foreign to them, not too important, shown by the following statements:

*“This is beyond my control”*

*“It is something that does not depend on me”.*

These emotional phases had occurred in all of them to a greater or lesser extent, moving easily forwards and backwards. On the other hand, women admitted that breast cancer was a stressful factor that caused emotional (anxiety, irritability, rage, guilt, etc.), cognitive (confusion, lack of concentration, forgetfulness, etc.) and even physiological disturbances.

#### 3.1.2. Theme 2: Emotional Isolation

They explained that they felt isolated and lonely. They were not able to identify clearly their immediate social environment during the illness. In addition, it was even more difficult to define their essential source of social and emotional support, probably because their relatives and friends did not know how to react to their disease. One of the most repeated sentences was the following statement:

*“There were many people who disappeared after knowing my diagnosis”.*

Their most immediate relationships were their husband/partner and children. They identified three types of partners. For the first one, most women described a man who decided to leave home, incapable of assuming cancer. However, they admitted that the breakup of the couple usually was the consequence of prior problems, demonstrated by the following statement:

*“If things were not going well until that moment, everything was destroyed with the disease”.*

A smaller number of women described a second type of husband, insensitive to women’s feelings, demonstrated by the following statement:

*“I can’t complain at home, because my husband behaves as if nothing happens. Then, I am trying to be the same person as always”.*

For a smaller group of women, there was a third type of partner who tried to take care for them. Nevertheless, two different subtypes were described in this case, the one who cared too much about the woman, with a mix of pity and parental protection. However, women admitted that they felt misunderstood, shown by the following statement:

*“He makes me even more nervous with that overprotection that it is not good for me”.*

The least usually described type of husband was one who faced cancer directly, without taboos, with an authentic and real understanding.

Regarding children, they were always a source of concern. They did not usually seek support from their children, but they tried to protect them. Two themes were recurrent, their concern for their future, especially in the early stages of cancer, and the fear that their daughters may inherit breast cancer, as shown in the following statements:

*“I only ask that this disease allows me to be with them until they are self-sufficient”.*

*“I wish that this disease finishes with me”.*

Children’s age was a critical factor, as young children and adolescents were of major concern and direct responsibility for women, while adult ones may support their mothers.

With respect to friends, they were mostly not aware of their disease because of the patient’s wish and the cruel curiosity of these friends, shown by the following statement:

*“There are people who don’t want to help, they just want to know”.*

Nevertheless, sometimes, they realized that there were people who were committed to women, willing to help and care for them.

#### 3.1.3. Theme 3: Lack of Information

In the focus groups, there was an agreement in three key elements. The first one was what women defined as “*the meaning of the search*”. They had tried to look for information about breast cancer, as they thought that information provided by health professionals was insufficient, shown by the following statement:

*“I needed to know the enemy to face it”.*

The second element was that most women felt responsible and guilty of their own illness, demonstrated by the following statement:

*“I have gotten into this, I have to go out”.*

The third idea had to do with social comparisons and they tended to compare their problem-solving skills with those of healthy women from their immediate social environment. However, as those women were not sick, their imitation would not help them against breast cancer.

#### 3.1.4. Theme 4: Inability to Give Advice on Breast Cancer to Other Women

Women admitted that they were still lost when focus groups were carried out, and were not able to give advice to other women. They frequently commented the following statements:

*“I have not yet discovered what to do with my life”.*

*“Who am I to advise?”.*

All of them agreed on the need to have an answer for all their doubts and fears. Having good information was basic to face any disease. The most important concerns for them were as follows:

*“Am I going to die?”*

*“How do I explain to my relatives what happens to me?”*

*“Why me? What does it depend on?”*

*“Will health professional help me enough? Are they telling me the truth?”*

*“What is the medical treatment? Will I keep my breast?”*

*“What are the sexual repercussions? Will my husband help me?”*

*“Will my family withstand this situation?”.*

Most of them highlighted the absence of communication with healthcare professionals, who failed to understand their needs and fears about breast cancer.

For most women, the most stressful moment was when they had to attend a new medical review. As there was an absolute absence of information or emotional support after having been discharged, feelings of general discomfort and loss of strength emerged with any check-up. They said the following statement:

*“The Sword of Damocles was hanging over my head”.*

## 4. Discussion

Focus groups were chosen to carry out this study, as we were working with women in which breast cancer diagnosis and treatment had changed their routine and habits, triggering a deeply disturbing situation [27]. Their needs’ satisfaction had been threatened, causing physical, psychological and social strains that their organism tried to delete by starting a set of physiological, cognitive, emotional and social reactions to adapt themselves to this new situation [28]. In this sense, focus groups help people to express and clarify their feelings and points of view through group interaction that are unlikely to occur with other methods, such as questionnaires or one-to-one interview [29]. We have observed that women had implemented defense mechanisms, such as denial, rationalization or projection [30]. Some of them had had difficulties in coping with all the changes; others decided to face the new situation by using those resources they had previously. Finally, only a few women learned to use new coping resources, emerging stronger from their disease.

Although several factors have been extensively studied in breast cancer, such as tumor types and prognosis, risk factors, symptomatology, diagnosis or treatment possibilities, the same interest has not been shown for the emotional world, especially after diagnosis or during the time of recovery [31,32]. In our study, most women reported medical reviews as one of the moments of greatest uncertainty, and the longer the interval between two check-ups, the less control they felt over their own health. However, other authors [33] described that the most stressful moment for breast cancer women was the period of time between diagnosis and the surgical intervention. Regardless of the most frustrating moment, it is clear that certain negative emotions associated with breast cancer are sustained for a long time after treatment completion (several months or even years). Many patients described having anxiety, fear of recurrence and feelings of loneliness, as well as social, physical, labor or sexual problems. They remained concerned about breast cancer even when the cancer had completely disappeared, probably because of the uncertainty of recurrence [34]. Thus, treatment completion does not imply the end of the adaptation process, but that it continues throughout the follow-up phase. In our study, a large number of women decided not to express their emotions to avoid harming their immediate social environment [35]. A high incidence of a potential repressive style in breast cancer survivors was also observed [36].

Regarding coping strategies and the relationship between cognitions, behaviors and emotional answers, we have observed, as other authors [37], that women had emotional control mechanisms to counteract the discomfort caused by an unpleasant emotion. In our study, most women carried out a repressive coping style and they tried to control each answer of a negative emotion, triggering a huge discordance among the physiological, cognitive and motor response systems. Consequently, a significant percentage of women expressed depressive or anxiety feelings. Depression and anxiety have also been reported in breast cancer women after diagnosis, as well as maladaptive reactions, affective inhibitions and high levels of psychological stress and anger [38].

In a previous study [39], we have observed, on the one hand, that breast cancer survivors show different patterns of long-term emotional needs and, on the other one, that psychological therapeutic interventions should be maintained in many patients over time, even after treatment completion. In the current study, we have tried to apply our results to women’s recovery. Other authors [40,41] have assessed the repressive coping style, or the existence of depression or anxiety, and their influence in the development of cancer, but they did not evaluate how these coping styles affect patient recovery. Focus groups have allowed us to verify that the coping style chosen by each woman may determine the recovery from their disease; if women choose a positive coping style, they may develop their emotional abilities to overcome breast cancer. By contrast, if a repressive coping style is carried out, women greatly limit their emotional tools, becoming dependent on external actions, and preventing their body from evolving towards full recovery.

Our final aim was to establish the most appropriate psychological intervention guidelines when treating these women. They do not only need treatment at a medical level, but also at a psychological one, taking into account the emotional state in which they are at a certain moment [42]. Several factors, such as genetic background, initial general condition or previous relationship with cancer, can alter their emotional answers to breast cancer. Thus, any psychological intervention may be individualized [43]. As Levkovich *et al.* reported [44], we have observed that diagnosis and treatment are accompanied by psychosocial problems in a large number of patients. In addition, healthcare systems overestimate technical procedures instead of using empathy, which should be present in any healthcare professional [45]. Although a multidisciplinary team should treat these women (doctors, psycho-oncologists, psychologists, nurses and nursing assistants), nurses play an essential supporting role for these women, as they are those healthcare professionals who spend more time with these patients. On the other hand, the nursing task should be addressed not only to patients but also to their relatives, trying to recognize their particular needs [46]. Thus, these tasks can be summarized by the following three points:

- Providing care to patients from a more humanistic point of view, also including their relatives.
- Ensuring that they have a high quality of life, in spite of breast cancer.
- Reducing the impact of hospital dynamics on people that are not used to health services.

Any breast cancer patient is susceptible to receive emotional supportive therapy by nurses, facilitating the expression of emotions and teaching the patient how to handle the problems associated with cancer [42]. Once emotional needs are identified, interventions should include the following [28]:

- Providing information to patients and families about their emotional state.
- Explaining them the best ways to share and show feelings within the family sphere.
- Teaching the patient how to detect negative feelings and handle them through emotional self-control techniques.

To be effective, these interventions should be carried out within a dynamic and progressive training program, from the most basic behaviors to the expression of feelings [6]. Regarding coping strategies and adaptation, a new assessment should be carried out, which should include the following [12]:

- Patient perception about themselves, their disease and mood.
- Coping resources, such as health resources, social and problem-solving skills, social support, economic and personal resources, and their relationship with healthcare personnel.
- Patient’s ability to overcome breast cancer.
- Physical and emotional signs of stress.

Healthcare professionals may help survivors to identify all those reactions they have in certain situations of their disease. Once aware of them, women will be able to recognize these stressful stimuli. The second intervention may be aimed at managing these negative stimuli. Then, women should learn how to correct or adapt themselves to these stimuli as healthy as possible. Patients should be taught that stressful stimuli can be controlled, but not eliminated in a radical way. Once addressed these objectives, health professionals can talk about the adaptive responses, as survivors have to learn and develop effective mechanisms for any situation triggered by the disease.

Breast cancer diagnosis and treatment are an important source of stress that triggers a wide variety of adaptation problems. Medical success is also related to the affective characteristics of patients, and the confidence generated by healthcare professionals is a powerful predictor for the evolution of the disease. Nurses are optimally positioned to address survivor specific emotional needs in order to develop proper emotional support programs. Focus groups may be a valuable tool for guiding the development of interventions by nurses. They may help women to express emotions and, subsequently, implement optimal coping strategies to improve their recovery.

The results of this study have implications for nursing practice. Therapeutic interventions performed by nurses should be carried out from the very moment of diagnosis and maintained and prolonged in time, in order to consolidate adaptive responses of a lasting nature. Health professionals cannot neglect that learning to handle emotions is essential to maintain and recover the quality of life in these patients [6]. The current specialization of healthcare professionals has, as a disadvantage, the possibility of compromising care due to the emphasis given to the disease and not to the person. It is crucial that nursing protects, improves and preserves humanity in care. Focus groups are one of the most appropriate approaches to establish an effective professional–patient relationship, as they can provide major insights into patient opinions, attitudes and beliefs. The final goal would be to reduce negative emotions and achieve comprehensive and holistic care for breast cancer survivors.

### Limitations

Our study has several limitations. As any qualitative study, the findings may be affected by the experience and perception of the research team, as well as the characteristics of the participants. Moreover, although focus groups yielded in-depth data that would not have been easily obtained by other methods, there may have been variations in the way that they were conducted by the researchers. On the other hand, self-reported data obtained through focus groups can rarely be verified, and may contain potential sources of bias. Finally, attribution or exaggeration bias may have occurred during the study.

## 5. Conclusions

The use of focus groups in women diagnosed with breast cancer allows nurses to evaluate the expression of their emotions, as well as to collect and share information about their feelings, thoughts and experiences, facilitating this process so that patients can cope with the stress related to their illness.

One of their main concerns was the lack of adequate information about breast cancer, which led to misconceptions about this disorder, the current state of their disease and their recovery. They have also expressed feelings of guilt. Likewise, most have emphasized the poor relationship with their family, their concern for their children and the risk that their daughters may inherit breast cancer.

## Figures and Tables

**Table 1:** Sociodemographic characteristics of the sample (*n* = 83).

	Number (%) of Patients
**Age** (years)	
30–39	1 (1.2%)
40–49	31 (37.3%)
50–59	33 (39.8%)
60–69	16 (19.3%)
70–79	2 (2.4%)
**Marital status**	
Married/with partner	47 (56.6%)
Widowed	4 (4.8%)
Single	11 (13.3%)
Separated/divorced	14 (16.9%)
No answer/do not know	7 (8.4%)
**Children**	
Yes	67 (80.7%)
No	16 (19.3%)
**Education**	
Elementary school	22 (26.5%)
Secondary school or similar	31 (37.3%)
Higher education	28 (33.7%)
No answer/do not know	2 (2.4%)
**Employment situation**	
Employed	39 (47.0%)
Unemployed	9 (10.8%)
Housewife	12 (14.5%)
Retired	13 (15.7%)
Temporary incapacity for work	5 (6.0%)
No answer/do not know	5 (6.0%)

**Table 2:** Clinical characteristics of the sample (*n* = 83).

	Number (%) of Patients
**Disease stage**	
I	5 (6.0%)
II	69 (83.1%)
III	6 (7.2%)
Cured	1 (1.2%)
No answer/do not know	2 (2.4%)
**Prognosis** (defined by patients)	
Good	25 (30.1%)
Bad	29 (34.9%)
Not defined	27 (32.5%)
No answer/do not know	2 (2.4%)
**Treatment**	
Chemotherapy	79 (95.2%)
Radiotherapy	62 (74.7%)
Endocrine therapy	30 (36.1%)
Surgery	79 (95.2%)
**Type of surgery**	
Tumorectomy	5 (6.0%)
Simple mastectomy	40 (48.2%)
Radical mastectomy	36 (43.4%)
**Current situation of disease**	
Without recurrence	43 (51.8%)
Follow-up after recurrence	28 (33.7%)
Active disease	8 (9.6%)
No answer/do not know	4 (4.8%)
**Interval between reviews**	
Three months	15 (18.1%)
Six months	24 (28.9%)
One year	22 (26.5%)
More than one year	16 (19.3%)
No answer/do not know	6 (7.2%)

## Data Availability

Data are available from the corresponding author on reasonable request.
